# Men, Women, and Ghosts in Science

**DOI:** 10.1371/journal.pbio.0040019

**Published:** 2006-01-17

**Authors:** Peter A Lawrence

## Abstract

Science suffers because, by favouring the self-confident of both sexes, we discriminate against women.

*At the current pace, European women are not expected to reach parity with men in academic science positions until 2050.* —Gerlind Wallon [1]

Some have a dream that, one fine day, there will be equal numbers of men and women in all jobs, including those in scientific research. But I think this dream is Utopian; it assumes that if all doors were opened and all discrimination ended, the different sexes would be professionally indistinguishable. The dream is sustained by a cult of political correctness that ignores the facts of life—and thrives only because the human mind likes to bury experience as it builds beliefs. Here I will argue, as others have many times before, that men and women are born different. Yet even we scientists deny this, allowing us to identify the “best” candidates for jobs and promotions by subjecting men and women to the same tests. But since these tests favour predominantly male characteristics, such as self-confidence and aggression, we choose more men and we discourage women. Science would be better served if we gave more opportunity and power to the gentle, the reflective, and the creative individuals of both sexes. And if we did, more women would be selected, more would choose to stay in science, and more would get to the top.

## A Taboo

It is not easy to write or talk about this subject. If you say, for example, that women are *on average* more understanding of others, this can be interpreted as misogyny in disguise. If you state that boys *on average* are much more likely than girls to become computer nerds, people may react as if you plan to ban all women from the trading rooms of merchant banks. The Cambridge University psychologist Simon Baron-Cohen published research on the “male brain” in a specialist journal in 1997, but did not dare to talk about his ideas in public for several years [2]. One reason for this absurd taboo is that we cannot think objectively because our minds are full of wayward beliefs and delusions—“ghosts” (Box 1). And one of these ghosts is the dogma that all groups of people, such as men and women, are *on average* the same, and any genetic distinctions must not be countenanced. Such ghosts bias our perceptions and censor our thoughts.

**Box 1. Ghosts**

“**Mrs. Alving:** I almost think we are all ghosts— all of us, Pastor Manders. It isn't just what we have inherited from the father and mother that walks in us. It is all kinds of dead ideas and all sorts of old and obsolete beliefs. They are not alive in us; but they remain with us none the less, and we can never rid ourselves of them. I only have to take a newspaper and read it, and I see ghosts between the lines. There must be ghosts all over the country. They lie as thick as grains of sand. And we're all so horribly afraid of the light” [3].

## Boys and Girls Are Born Different and Remain So

*The chance that a woman will mug you tonight on the way home is somewhere around nil. That is a quirk specific to my gender.* —Michael Moore [4]

Baron-Cohen makes one point crystal clear: you cannot deduce the psychological characteristics of any person by knowing their sex. Arguing from the scientific literature that men and women typically have different types of brains, he nevertheless points out that “some women have the male brain, and some men have the female brain” [2]. Stereotyping is unscientific—“individuals are just that: individuals” [2]. Yet Baron-Cohen presents evidence that males *on average* are biologically predisposed to systemise, to analyse, and to be more forgetful of others, while females *on average* are innately designed to empathise, to communicate, and to care for others. Males tend to think narrowly and obsess, while females think broadly, taking into account balancing arguments. Classifying individuals in general terms, he concludes that among men, about 60% have a male brain, 20% have a balanced brain, and 20% have a female brain. Women show the inverse figures, with some 60% having a female brain. Many facts (see [2] for references) argue that these differences have their roots in biology and genetics. Here are some examples. First, it is hardly necessary to point out that distinguishing between the contributions of nature and nurture to animal or human behaviour has proved difficult. However, newborn infants (less than 24 hours old) have been shown a real human face and a mobile of the same size and similar colour. *On average*, boys looked longer at the mobile and girls looked longer at the face [5]. Second, such differences at birth must have developed earlier. One factor is the level of testosterone in the developing brain around three months of gestation, which is higher in males (due to the hormone being produced by the foetus itself). Many studies show that testosterone affects development and behaviour, not only in humans, but also in other mammals. Testosterone sponsors development of the male phenotype, and can influence behaviour even of animals of the same sex. For example, giving older men testosterone specifically improves their ability with those spatial tests on which males normally score higher than females [6].

Third, autism spectrum conditions are genetically based, and have been described in detail [2,7]. People with these problems communicate poorly; they are unable to put themselves in another's place, and have difficulties with empathising. They may treat others as objects. They often become obsessed and show repetitive behaviour. The less severely affected can become experts on recondite subjects, such as train timetables or ocean temperatures. Most relevant for our arguments is that autism spectrum conditions are largely sex-limited, being between four and nine times more frequent in males. From many studies, including psychology and neuroanatomy, Baron-Cohen argues convincingly that autism spectrum conditions are an extreme form of maleness [2,8].

It will not have escaped the notice of many scientists that some of their colleagues and maybe themselves have more than a hint of these “autistic” features. There is good evidence that this type of single-mindedness is particularly common in males [2]. Indeed, we might acknowledge that a limited amount of autistic behaviour can be useful to researchers and to society—for example, a lifetime's concentration on a family of beetles with more than 100,000 species may seem weird, but we need several such people in the world for each family. And most of these specialists will be men. (The Web pages of the Smithsonian Institute in Washington suggest that their systematists consist of about 30 women and 125 men.) It follows that if we search objectively for an obsessive knowledge, for a mastery of abstruse facts, or for mechanical understanding, we will select many more men than women. And if males *on average* are constitutionally better suited to be this kind of scientist, it seems silly to aim at strict gender parity.

However, in professions that rely on an ability to put oneself in another's place, at which women *on average* are far superior, we should expect and want a majority of women. For example, among current student members of the British Psychological Society, there are 5,806 women to 945 men; and among graduate psychologists, 23,324 women to 8,592 men. Of those who practice as chartered psychologists, the ratio has fallen further (7,369 women to 4,402 men). Yet among Fellows of the Society, honoured largely for their research, there are 428 men to only 106 women!

## Representation of Men and Women in Science

Among biomedical students in Europe and in the United States, there are similar numbers of males and females, suggesting perhaps that this subject is equally well suited to both sexes. But with higher and higher rank, the proportion of women falls inexorably—full professors are only about 10% female [9]. Women drop out steadily, and many of them have demonstrated high ability. There is plenty of evidence for similar trends in different branches of science [9]. For example, at the Laboratory of Molecular Biology in Cambridge, UK, where I work, the gender ratio of graduate students is currently 43 male to 35 female, yet the ratio of group leaders is 56 male to 6 female.

Science would be better served if we gave more opportunity and power to the gentle, the reflective, and the creative individuals of both sexes.

Are there social or practical reasons why we would like to maintain a more equal balance, especially where scientists have power over others? The short answer is yes, and here are three reasons:

First, these top research jobs call for a mix of skills, which a mix of men and women can deliver best. Nowadays, holders of these jobs plan science projects, write grants and articles, and try to network their papers into the top journals. Their students and postdocs, mostly young and inexperienced, usually do all the bench work. These students need more than instructions; they also need mentors who are able to listen to them and teach them understandingly. Indeed, some individuals deserve freedom to work out their own ideas: for example, Einstein did not have his doctorate when he wrote four of six of his great papers. Not many students get such opportunities now—whatever their potential. Understanding individuals and working out how to make the best of their diverse abilities are, as we have seen, predominantly feminine qualities.

Second, if we had a balanced mix of men and women in charge of our institutes, I believe we would have more contented and productive workplaces. We should not forget that the motivation to work hard and solve problems can come from supportive colleagues, as well as from competitiveness.

Third, it is self-evident that scientific leaders should include a diversity of people from whom younger individuals can pick role models as they choose their careers. The present lack of top female scientists will divert young women from scientific ambition; it makes no sense to discourage a future Frances Crick.

Many have turned their attention to explaining the fall out of women from science; it is traditionally ascribed to a mixture of discrimination and choice [9]. Regarding overt discrimination, in a lifetime in science, I have seen only little, and it has been both for and against women. Surely, gender discrimination cannot explain more than a tiny part of this trend. However, choice is certainly a main factor. Some choices are unavoidable; if there are to be children, women must bear them. However, after about six months or so, there is no reason, in principle, why the main carer of the children should not be the father. Later on, it could just as well be the father who takes time off work to look after a sick child. Yet partly because of the different priorities that *on average* men and women have, a much higher proportion of women put the needs of their children first and climbing the career ladder second.

But there is a different kind of discrimination that particularly damages creative pursuits such as science. There is good psychological evidence that aggression and lack of empathy are *on average* male characteristics, and we may agree with Baron-Cohen that for both sexes, “nastiness…. gets you higher socially, and gets you more control or power” [2,10,11]. Science should not be a military or a business operation, but nowadays it increasingly resembles one—for most, it is a vicious struggle to survive. In this struggle, men climb higher because they are *on average* more ruthless, and many women, as well as a gentle minority of men, shy away from competing with them [12]. And I think that our selection methods exacerbate this tendency.

## Job Searches in Academia

About 100 years ago, Ibsen shed light on the secrets of contemporary life, and in doing so, championed women's rights. But since then, the feminist campaign for equality has helped build the belief that men and women, *on average*, have exactly the same aptitudes. It is time we exorcised this particular ghost, and if we do, it will help put more of the less aggressive members of society, most of whom are women, into positions of power. For example, in job searches and in considering people for promotions, we have been asking women to take tests, largely devised by men, that tend to overvalue masculine characteristics. If men and women *on average* were identical, no one would see fault in this, but if it is agreed that they are not, these tests become discriminatory—for they favour those many men and those few women with masculine behaviour.

At present, in the competition for academic posts, we expect our candidates to go through a gruelling process of interview that demands self-confidence. We are impressed by bombast and self-advertising, especially if we don't know the field, and we may not notice annexation of credit from others, all of which *on average* are the preferred province of men. But we should also seek out able scientists who would care well for their groups, those who would mentor a distressed student and help her or him back into productive research. And if we did, we would choose more feminine women as well as more feminine men.

And most important of all, could we try to select for the one characteristic we need most, scientific originality? Originality and creativity are all too rare, and I know of no evidence that these traits are more frequent in one sex [13]. As we busily compare candidates, adding up their papers and calculating impact factors, do we remember to look for these qualities? Instead of reading the papers, we count them. Counting rewards those who have had many papers accepted, and those who have worked their names into the author list. But is the editorial process of selecting papers an objective one? Certainly not; in the jungle where we fight to publish, salesmanship and pushiness pay off [14], and these tend to be masculine characteristics. Thus, if we were to read the papers of candidates and search for originality and insight, I believe we would select more women, as well as more men with feminine qualities. So I am not advocating overt positive discrimination; instead, I suggest we consciously try to see through showmanship and select the qualities we actually need.

I have argued that reducing the premium we give to aggression would, in several different ways, lead to more women in science and also to better science. Even so, in this Utopia, I think that far less than 50% of top physicists would be women (and far less than 50% of top professors of literature would be men). But I don't think that would matter—we would be making better use of the diverse qualities of people. Both women and men might accept that although there is much overlap in the two populations, we are constitutionally different—a diversity we should be able to celebrate and discuss openly. Both women and men should be leading such discussions with pride.

## References

[pbio-0040019-b1] Schubert C, Sinha G (2004). A lab of her own. Nat Med.

[pbio-0040019-b2] Baron-Cohen S (2003). The essential difference. Men, women and the extreme male brain.

[pbio-0040019-b3] Ibsen H, Meyer M (2005). Ghosts.

[pbio-0040019-b4] Moore M (2004). Dude where's my country.

[pbio-0040019-b5] Connellan J, Baron-Cohen S, Wheelwright S, Ba'tki A, Ahluwalia J (2001). Sex differences in human neonatal social perception. Infant Behav Dev.

[pbio-0040019-b6] Janowsky JS, Oviatt SK, Orwoll ES (1994). Testosterone influences spatial cognition in older men. Behav Neurosci.

[pbio-0040019-b7] Frith U (2003). Autism: Explaining the enigma (cognitive development). 2nd edition.

[pbio-0040019-b8] Baron-Cohen S, Knickmeyer RC, Belmonte MK (2005). Implications for explaining autism. Science.

[pbio-0040019-b9] European Technology Assessment Network Working Group on Women and Science (2000). Science policies in the European Union: Promoting excellence through mainstreaming gender equality.

[pbio-0040019-b10] Savin-Williams RC (1987). Adolescence: An ethological perspective.

[pbio-0040019-b11] Chekhov AP, Frayn M (1983). The three sisters.

[pbio-0040019-b12] Babcock S, Laschever S (2004). Women don't ask: Negotiation and the gender Divide.

[pbio-0040019-b13] Alpaugh PK, Birren JE (1975). Are there sex differences in creativity across the adult life span?. Hum Dev.

[pbio-0040019-b14] Lawrence PA (2003). The politics of publication. Nature.

